# ﻿Systematic notes on three troglobitic *Anapistula* (Araneae, Symphytognathidae) spiders from China, with the descriptions of two new species

**DOI:** 10.3897/zookeys.1130.91467

**Published:** 2022-11-21

**Authors:** Shuqiao Wang, Ying Lu, Ya Li, Shuqiang Li, Yucheng Lin

**Affiliations:** 1 Key Laboratory of Bio-resources and Eco-environment (Ministry of Education), College of Life Sciences, Sichuan University, Chengdu 610065, China; 2 The Sichuan Key Laboratory for Conservation Biology of Endangered Wildlife, Sichuan University, Chengdu, Sichuan 610064, China; 3 Institute of Zoology, Chinese Academy of Sciences, Beijing 100101, China

**Keywords:** Cave spider, description, molecular analysis, symphytognathids, taxonomy

## Abstract

Three cave-dwelling spider species belonging to the family Symphytognathidae Hickman, 1931, i.e., *Anapistulasanjiao***sp. nov.** (♂♀), *A.walayaku***sp. nov.** (♂♀), and *A.panensis* Lin, Tao & Li, 2013 (♂♀), are reported from southwest China. DNA sequences and detailed illustrations of the habitus, male palps and epigynes are provided, and their distributions are mapped. Their phylogenetic position within symphytognathids and relationships were tested and assessed using previously published phylogenetic analyses on symphytognathoids. The results showed that they form a clade with *A.choojaiae* Rivera-Quiroz, Petcharad & Miller, 2021 from Thailand.

## ﻿Introduction

The genus *Anapistula* Gertsch, 1941 includes 26 described species. It is the second-most speciose genus of the Symphytognathidae Hickman, 1931, with more than half of the species widespread in the tropical and subtropical regions of the Oriental and Neotropical realms (WSC 2022). Eighteen known *Anapistula* species have been collected from the leaf litter, soil or mosses (Gertsch 1941; Forster 1958, 1959; Forster and Platnick 1977; Baert and Jocqué 1993; Saaristo 1996; Harvey 1998; Ono 2002; Rheims and Brescovit 2003; Tong and Li 2006; Rubio and González 2010; Lin et al. 2013; Dupérré and Tapia 2017; Rivera-Quiroz et al. 2021), seven live in caves (Harvey 1998; Rheims and Brescovit 2003; Cardoso and Scharff 2009; Lin et al. 2009; Lin et al. 2013), and only one was found at a cave entrance (Rheims and Brescovit 2003).

The type species, *Anapistulasecreta* Gertsch, 1941, is widely distributed from the USA to Colombia, the Bahamas and Jamaica (Dupérré and Tapia 2017). The first described Asian *Anapistula* species is *A.jerai* Harvey, 1998 from Malaysia and Indonesia (Harvey 1998). Additional Asian species include *Anapistulaappendix* Tong & S. Li, 2006 (China), *Anapistulachoojaiae* Rivera-Quiroz, Petcharad & Miller, 2021 (Thailand), *Anapistulaishikawai* Ono, 2002 (Japan), *Anapistulaorbisterna* Lin, Pham & S. Li, 2009 (Vietnam), *Anapistulapanensis* Lin, Tao & Li, 2013 (China), *Anapistulazhengi* Lin, Tao & Li, 2013 (China) (Ono 2002; Tong and Li 2006; Lin et al. 2009; Lin et al. 2013; Rivera-Quiroz et al. 2021). Considering the two new species described here, the genus now consists of 28 species, half of which are described only from one sex. There are four species represented only by males, and 10 species in which only females are known.

The aims of this paper are: 1) to report three cave-dwelling *Anapistula* species from China, two of them new to science, and 2) to verify their sex pairing and resolve their phylogenetic relationships within symphytognathids. We used a combination of newly generated sequences and others available from GenBank to build a molecular phylogeny of the Symphytognathidae to confirm the generic placement of our new species.

## ﻿Materials and methods

### ﻿Specimen sampling

Specimens studied here were collected from caves in Yunnan and Guizhou provinces, in southwest China, on or during 26 April 2010, 24 June 2016, 10–24 August 2018, and 24 August 2020. All of the specimens were captured by hand and stored in 95% ethanol at –20 °C.

### ﻿Molecular data

To test relationships within symphytognathids and the taxonomic position of the three *Anapistula* species, eight individuals were selected from the examined materials for molecular data collection. Their legs and prosoma were used to extract genomic DNA and sequence five gene fragments: 16S, 18S, 28S, COI and H3. The abdomens and male palps were kept as vouchers. All of the molecular data were obtained from specimens collected at the type localities, although not from the type specimens themselves. Whole genomic DNA was extracted from tissue samples with the TIANamp Micro DNA Kit (TIANGEN) following the manufacturer’s protocol for animal tissue. The five gene fragments were amplified in 25μL reactions. Primer pairs and PCR protocols are given in Table 1. Raw sequences were edited and assembled using BioEdit v.7.2.5 (Hall 1999). New sequences from this study were deposited in GenBank (Table 2). All molecular vouchers and examined materials are stored in the Natural History Museum of Sichuan University (NHMSU), China.

**Table 1.** The loci, primer pairs, and PCR protocols used in this study.

Locus	Annealing temperature/time	Direction	Primer	Sequence 5’→3’	Reference
16S	46.45 °C/30 s	F	16sb2_12864	CTCCGGTTTGAACTCAGATCA	Hormiga et al. 2003
		R	LR-J-13360	GTAAGGCCTGCTCAATGA	Feng et al. 2019
	47 °C/30 s	F	16S-A	CGCCTGTTTATCAAAAACAT	Palumbi et al. 1991
		R	16S-B	CTCCGGTTTGAACTCAGATCA	
18S	52.1 °C/30 s	F	18s_1F	TACCTGGTTGATCCTGCCAGTAG	Giribet et al. 1996
		R	18s_1000R	GTGGTGCCCTTCCGTCAATT	Balczun et al. 2005
28SD2	54.9 °C/30 s	F	28sa	GACCCGTCTTGAAACACGGA	Rix et al. 2008
		R	LSUR	GCTACTACCACCAAGATCTGCA	
COI	48.95 °C/30 s	F	LCO1490	GGTCAACAAATCATAAAGATATTGG	Folmer et al. 1994
		R	HCO2198	TAAACTTCAGGGTGACCAAAAAATCA	
	46 °C/30 s	F	LCO1490	GGTCAACAAATCATAAAGATATTGG	Simon et al. 1994
		R	COI-Nancy	CCCGGTAAAATTAAAATATAAACTTC	
H3	48 °C/30 s	F	H3af	ATGGCTCGTACCAAGCAGACVGC	Colgan et al. 1998
		R	H3ar	ATATCCTTRGGCATRATRGTGAC	
	50 °C/30 s	F	H3nf	ATGGCTCGTACCAAGCAGAC	
		R	H3nr	ATRTCCTTGGGCATGATTGTTAC	

**Table 2.** GenBank accession numbers for new DNA sequence data from three *Anapistula* species.

Species	Identifier	Sex/Stage	16S	18S	28S	COI	H3
*Anapistulapanensis*	HA020	♀/adult	–	OP120815	OP120929	OP117477	OP131579
	HA020	♂/juvenile	–	OP120816	OP120930	OP117478	OP131580
*Anapistulasanjiao* sp. nov.	HA125	♂/adult	–	OP120819	OP120933	OP117481	OP131583
	HA125	♀/adult	–	OP120818	OP120932	OP117480	OP131582
*Anapistulawalayaku* sp. nov.	HA138	♂/adult	OP133563	OP120822	OP120936	OP117484	OP131586
	HA138	♀/adult	–	OP120820	OP120934	OP117482	OP131584
	HA138	♀/juvenile	OP133562	OP120821	OP120935	OP117483	OP131585
	HA106	♀/adult	OP133561	OP120817	OP120931	OP117479	OP131581

We used these sequences and a selection from previously sequenced taxa to assemble a phylogeny of symphytognathid spiders. In total, 50 species of symphytognathoids representing the families Theridiosomatidae, Mysmenidae, Anapidae, and Symphytognathidae were used. Two tetragnathid species were used as outgroups. We used the MAFFT v.7.450 online server (https://mafft.cbrc.jp/alignment/server/) with default parameters to align the sequences of the three Chinese *Anapistula* species. All sequences were concatenated in Sequence Matrix v.1.7.8 (Vaidya et al. 2011). We used PartitionFinder2 (Lanfear et al. 2017) to identify the best-fit models of molecular evolution for each locus. GTR+I+G was selected for COI, H3, 18S and 28S, and GTR+G was selected for 16S.

The maximum parsimony (MP) tree was constructed using MEGA X (Kumar et al. 2018) with TBR (Tree-Bisection-Reconnection) branch swapping and 2000 bootstrap replicates with default parameters. Bayesian phylogenetic inference (BI) was performed using MrBayes v.3.2.7 (Ronquist et al. 2012) through the Cipres Science Gateway (Miller et al. 2010) using four Markov Chain Monte Carlo (MCMCs) chains with default heating parameters for 50,000,000 generations or until the average standard deviation of split frequencies was less than 0.01. The Markov chains were sampled every 1000 generations, and the first 25% of sampled trees were burn-in. The program Tracer v.1.7.1 (Rambaut et al. 2018) was used to analyse the performance of our BI analyses.

### ﻿Morphological data

Specimens were studied in ethanol using a Leica M205 C stereomicroscope. Habitus and copulatory organs were photographed with a Canon EOS 60D wide zoom digital camera (8.5 megapixels) mounted on an Olympus BX 51 compound microscope. Male palps and epigynes were examined after dissection and treated with lactic acid before being embedded in Hoyer’s Gum and placed on an ultra-thin slide to take photos of the dorsal and ventral sides. The digital photos were montaged using Helicon Focus v.3.10 (Khmelik et al. 2006) image stacking software. All measurements are in millimetres. Leg measurements are given as follows: total length (femur, patella, tibia, metatarsus, tarsus).

Nomenclature of the genital structures was based on Dupérré and Tapia (2017) and Rivera-Quiroz et al. (2021) for *Anapistula*. Abbreviations in the text and figures are as follows:

#### Male palp

**Co** conductor;

**C1** anterior projection of conductor;

**C2** posterior projection of conductor;

**Cy** cymbium;

**E** embolus;

**Pa** palpal patella;

**Sd** sperm duct;

**Te** palpal tibia.

#### Epigyne

**A** epigynal atrium;

**MD** median duct of vulva;

**Fd** fertilization duct;

**Lb** lateral branch of the MD;

**Llb** distal lobe of lateral branch;

**S** spermatheca.

#### Institutional acronyms

**IZCAS**Institute of Zoology, Chinese Academy of Sciences, Beijing, China;

**NHMSU** Natural History Museum of Sichuan University, Chengdu, China.

## ﻿Results

### ﻿Phylogenetic analysis

**Figure 1.**
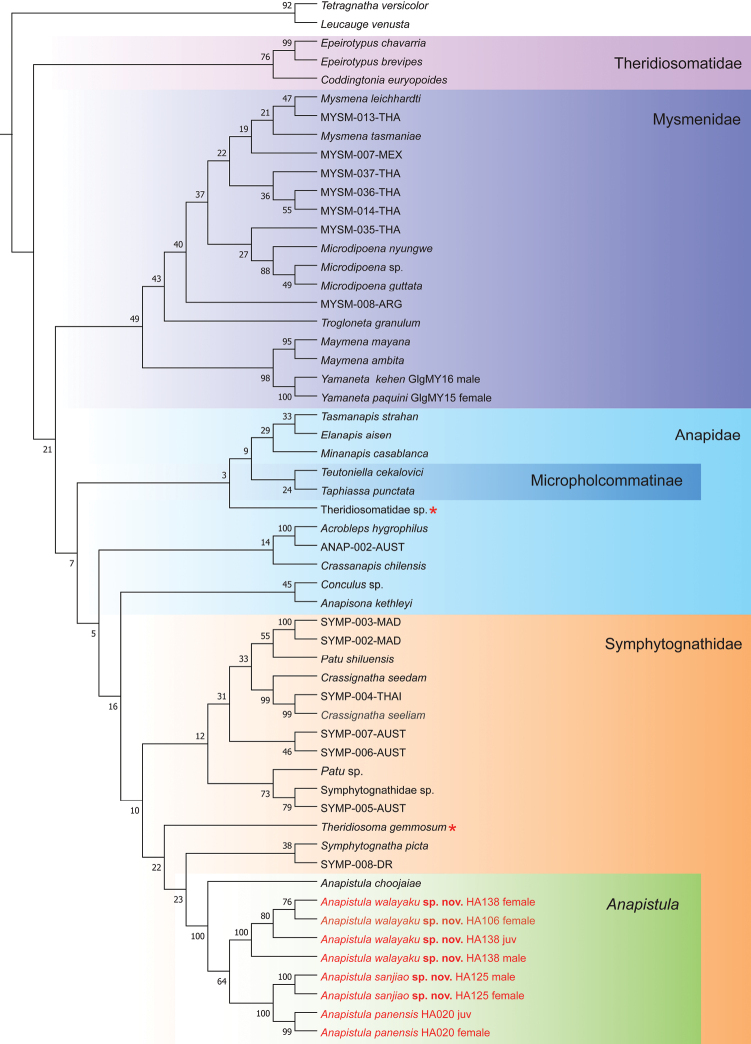
Tree topology obtained by maximum parsimony in MEGA-X using a modified version of Rivera-Quiroz et al. (2021) plus three Chinese *Anapistula* species (red font). Numbers at nodes indicate bootstrap support. Symphytognathidae is in the orange box and *Anapistula* in the green box. Note the paraphyly of Anapidae, the high support of *Anapistula* in Symphytognathidae, and the placement of Theridiosomatidae sp. (red star) within Anapidae and *Theridiosomagemmosum* (red star) within Symphytognathidae.

The MP analysis of the full dataset recovered a single most parsimonious tree topology (Fig. 1). This tree shows symphytognathoids are monophyletic but with low support. Theridiosomatidae, Mysmenidae and Symphytognathidae are monophyletic, also with low support. Here we note that *Theridiosomagemmosum* (L. Koch, 1877) (indicated by a red in the orange box in Fig. 1) is nested within the Symphytognathidae. Anapidae contains the polyphyletic Micropholcommatinae and an undescribed Theridiosomatidae species (indicated by a red star in the blue box in Fig. 1). A clade composed of four *Anapistula* species (three species in red font and *A.choojaiae* in Fig. 1) were highly supported as monophyletic. These results support our taxonomic classification.

**Figure 2.**
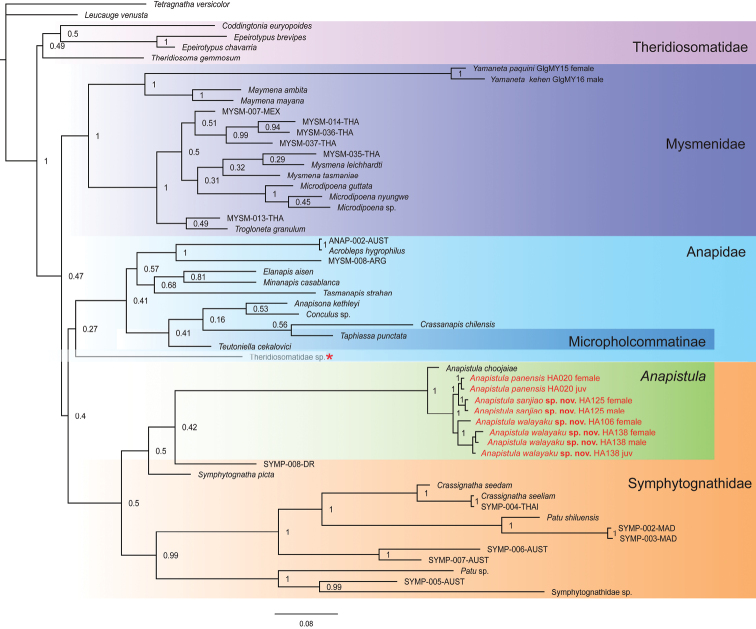
Tree topology from Bayesian analysis. Numerical values at nodes indicate posterior probabilities; other conventions as in Fig. 1. Note the high support of *Anapistula* in Symphytognathidae, and the monophyly of four *Anapistula* species; the low support of Anapidae, the nesting of Micropholcommatinae, and the placement of Theridiosomatidae sp. (red star) sister to Micropholcommatinae.

The result of BI is consistent with MP for some major clades, but there are some differences (Fig. 2). In the BI tree, Mysmenidae is highly supported compared to Theridiosomatidae, Anapidae and Symphytognathidae. However, an undescribed Theridiosomatidae species (marked by a red star in the blue box of Fig. 2) occurs between Anapidae and Symphytognathidae, and Micropholcommatinae is nested in Anapidae. Three Chinese and one Thai *Anapistula* species form a separate, highly supported clade in the BI topology. As a sister group, the clade of *Anapistula* is delimited to include: (*A.choojaiae* (*A.walayaku* sp. nov. (*A.sanjiao* sp. nov. + *A.panensis*))). Both the MP and BI analyses found the three Chinese and one Thai *Anapistula* species formed a clade sister to the remaining symphytognathids. The available molecular evidence seems sufficient to justify the taxonomic placement of the two new and one known *Anapistula* species.

### ﻿Taxonomy

**Family Symphytognathidae Hickman, 1931**

#### ﻿Genus Anapistula

Taxon classificationAnimaliaAraneaeSymphytognathidae

Gertsch, 1941

57687496-1369-515F-9114-466FCFD3CE99

Anapistula Gertsch, 1941: 2.

##### Type species.

*Anapistulasecreta* Gertsch, 1941 by original designation, from the Bahamas.

##### Diagnosis.

*Anapistula* differs from other genera of Symphytognathidae by: the presence of four lateral eyes in diads (most common) or the eyes are reduced to indistinct spots or absent (median eyes present in *A.boneti* Forster, 1958: figs 15, 16); the chelicerae are fused near the base, with two promarginal teeth; the cephalic area is slightly raised (strongly raised in *A.boneti*); a smooth carapace; and a sub-spherical abdomen without a colulus. Males are diagnosed by lacking clasping spines on tibia II, a cymbium without teeth or denticles but with long setae and apical lobes, a conductor, a short embolus (length less than ½ the diameter of the bulb), and a sperm duct coiled ca 1.5 times. Females are diagnosed by lacking palps, round spermathecae connected by a T- or Y-shaped epigynal median duct, and the absence of a scape and parmula (see Forster and Platnick 1977: fig. 19; Harvey 1998: figs 2, 4, 9, 14, 19; Dupérré and Tapia 2017: fig. 33; Rivera-Quiroz et al. 2021: figs 8d, 9c).

##### Composition in Asia.

*Anapistulaappendix* (♀, China), *A.choojaiae* (♂♀, Thailand), *A.ishikawai* (♀, Japan), *A.jerai* (♂♀, Malaysia, Borneo, and Indonesia), *A.orbisterna* (♀, Vietnam), *A.panensis* (♂♀, China), *A.sanjiao* S. Li & Lin, sp. nov. (♂♀, China), *A.walayaku* S. Li & Lin, sp. nov. (♂♀, China), and *A.zhengi* (♂♀, China).

##### Distribution in Asia.

China (Hainan, Guizhou, and Yunnan), Japan, Vietnam, Thailand, Malaysia, Borneo and Indonesia.

#### ﻿ Anapistula sanjiao

Taxon classificationAnimaliaAraneaeSymphytognathidae

S. Li & Lin sp. nov.

467C840D-1175-5677-B3C5-8407CCBA42D4

https://zoobank.org/7A23C5A8-EB67-46B6-A26E-D0C18CE53952

**Figure 3.**
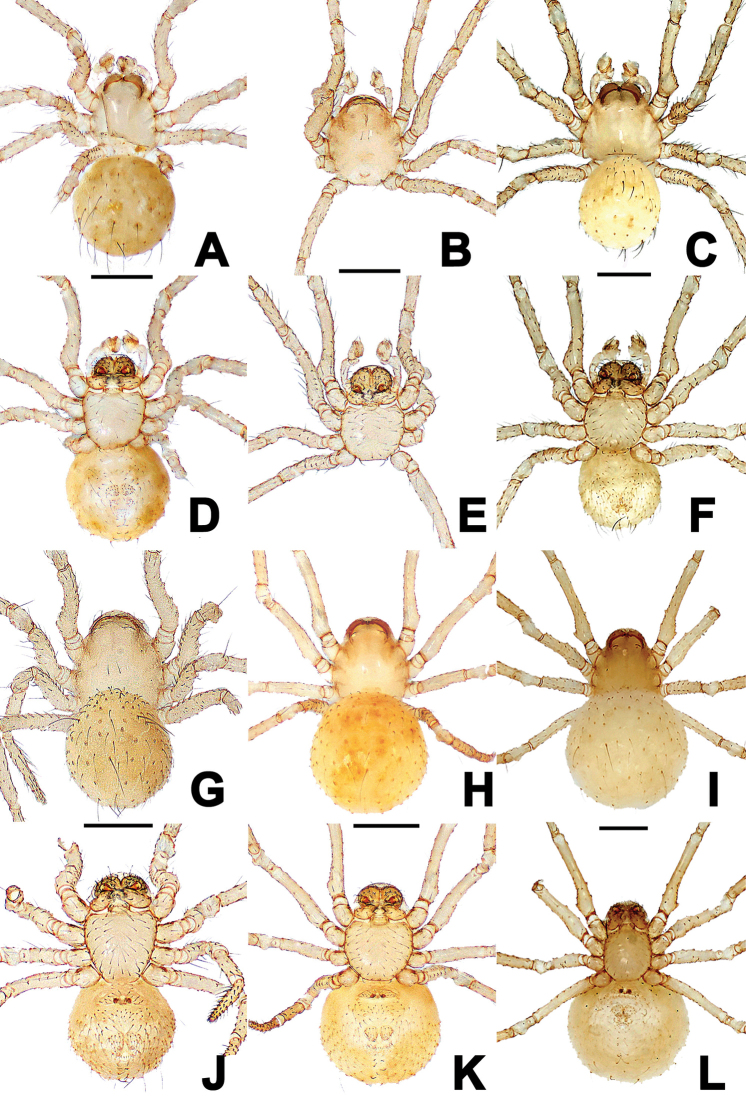
*Anapistulasanjiao* sp. nov. (**A, D, G, J**), *Anapistulawalayaku* sp. nov. (**B, E, H, K**), and *Anapistulapanensis* (**C, F, I, L**) **A, C** male habitus, dorsal **D, F** male habitus, ventral **B** male prosoma, dorsal **E** male prosoma, ventral **G–I** female habitus, dorsal **J–L** female habitus, ventral. Scale bars: 0.20 (**A–L**).

**Figure 4.**
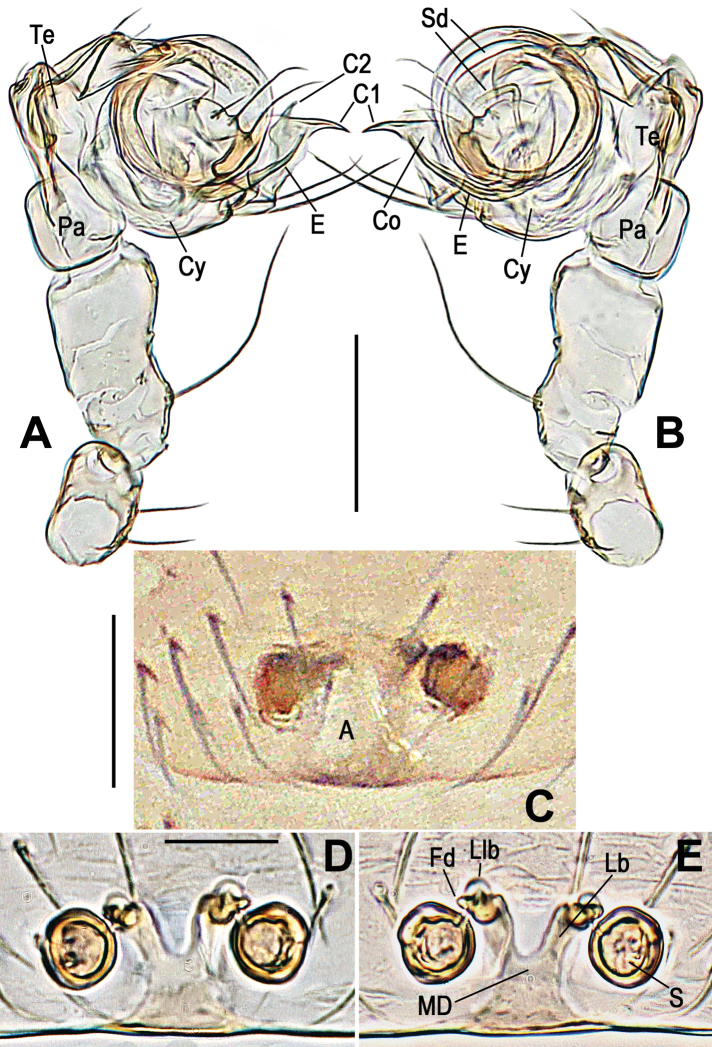
*Anapistulasanjiao* sp. nov. **A** male palp, prolateral **B** male palp, retrolateral **C** epigyne, ventral **D** vulva, ventral **E** vulva, dorsal. Abbreviations: A = epigynal atrium; Co = conductor; C1 = anterior projection of conductor; C2 = posterior projection of conductor; Cy = cymbium; E = embolus; Fd = fertilization duct; Lb = lateral branch of the MD; Llb = distal lobe of lateral branch; MD = median duct of vulva; Pa = palpal patella; S = spermatheca; Sd = sperm duct; Te = palpal tibia. Scale bars: 0.10 (**A–E**).

Figs 3A, D, G, J , 4A–E , 7A–D

##### Type material.

***Holotype*** ♀ and ***paratypes*** 1♂ 2♀ (NHMSU-HA125), **China**: Yunnan Province, Kunming City, Yiliang County, Jiuxiang Township, Dazhezong Village, Sanjiao Cave (25.13439°N, 103.39932°E, 1833 m), 24.VIII.2018, Y. Lin, Y. Li & Y. Shu leg.; 1♂ and 1♀ (NHMSU-HA125) used for sequencing, GenBank accession numbers given in Table 2, same data as for preceding.

##### Etymology.

The new species is named after the type locality; noun.

##### Diagnosis.

The male of this new species is similar to that of *A.zhengi* in the overall shape of the palp and in having C1 and C2 roughly as sharp as *A.zhengi* but differs in the length of C1 with respect to C2 and the presence of a small median projection between C1 and C2 (cf. Figs 4A, 7A to Lin et al. 2013: figs 6, 7). The female can be distinguished from most *Anapistula* species by the Y-shaped MD and its width greater than half the diameter of one spermatheca. The configuration of the vulva of *Anapistulasanjiao* sp. nov. seems most similar to that of *A.choojaiae* but differs by the smaller size of the spermathecae compared to the width of the MD, and the ends of the Llb are located beyond the anteromargin of the spermathecae (cf. Figs 4E, 7D to Rivera-Quiroz et al. 2021: fig. 9c).

##### Description.

**Male**: carapace ovoid, pale yellow with smooth surface and two central short setae (Fig. 3A). Lateral eyes degenerated into white eyespots, almost invisible (Fig. 3A). Chelicerae with two promarginal teeth. Labium rectangular, fused to sternum (Fig. 3D). Sternum peltate, slightly longer than wide, smooth, slightly convex, with sparse short setae, truncated posteriorly (Fig. 3D). Legs same colour as carapace. Abdomen sub-spherical, darker than prosoma and legs, cuticle with sparse long setae and weakly sclerotized patches (Fig. 3A, D). Spinnerets and anal tubercle pale yellow. Somatic measurements: body length 0.58, carapace 0.28 long, 0.24 wide, 0.20 high; sternum 0.20 long, 0.18 wide; length of legs: I 0.90 (0.24, 0.08, 0.22, 0.14, 0.22), II 0.76 (0.18, 0.10, 0.12, 0.10, 0.18), III 0.66 (0.12, 0.08, 0.16, 0.10, 0.20), IV 0.86 (0.22, 0.10, 0.20, 0.16, 0.18); leg formula I-IV-II-III; abdomen 0.35 long, 0.34 wide, 0.38 high.

***Palp***: weakly sclerotized (Figs 4A, B, 7A, B). Femur long, ca 2.2× length of patella, slightly constricted in ventral middle. Patella nearly cubic. Tibia oblate, ca 1.4× length of patella. Cymbium wraps around bulb prolaterally, with long setae at distal margin (Figs 4A, 7A). Sheath like conductor divided into two distal, sharp projections (C1 and C2 in Figs 4A, B, 7A, B); C1 longer than C2. Sperm duct (Sd) completes ca 1.8 loops in the bulb. Embolus sharp, protrudes from lower retrolateral edge of bulb, extends to retrolateral side of C1. End of embolus does not extend beyond CI. Embolic end no exceeds the C1 (Figs 4A, B, 7A, B).

**Female**: habitus as in male, except without palps (Fig. 3G, J). Somatic measurements: body length 0.60, carapace 0.28 long, 0.25 wide, 0.24 high; sternum 0.22 long, 0.20 wide; length of legs: I 1.10 (0.32, 0.12, 0.24, 0.20, 0.22), II 0.86 (0.26, 0.10, 0.20, 0.12, 0.18), III 0.76 (0.22, 0.08, 0.16, 0.12, 0.18), IV 0.88 (0.20, 0.10, 0.24, 0.16, 0.18); leg formula I-IV-II-III; abdomen 0.36 long, 0.0.32 wide, 0.40 high.

***Epigyne***: flat, without scape. Internal structures faintly visible via cuticle (Fig. 4C). Atrium long, subtriangular. Spermathecae spherical, strongly sclerotized relative to rest of body (Fig. 3J). MD as wide as diameter of spermatheca (Figs 4E, 7C). Lb diverging from the MD, forming a “Y” shape (Figs 4E, 7D). Lb as long as MD, wide as ca ½ of MD. Llb small, nodular, at distal end of Lb (Figs 4E, 7D).

##### Natural history.

The species lives in the crevices of cave entrance walls and in rubble on the cave floor.

##### Distribution.

China (Yunnan) (Fig. 10).

#### ﻿ Anapistula walayaku

Taxon classificationAnimaliaAraneaeSymphytognathidae

S. Li & Lin sp. nov.

3328FD66-CCF7-5490-B7E5-F2C2F425A752

https://zoobank.org/2AC52BB0-C4EB-49BE-80B6-0324F4FD7BEC

**Figure 5.**
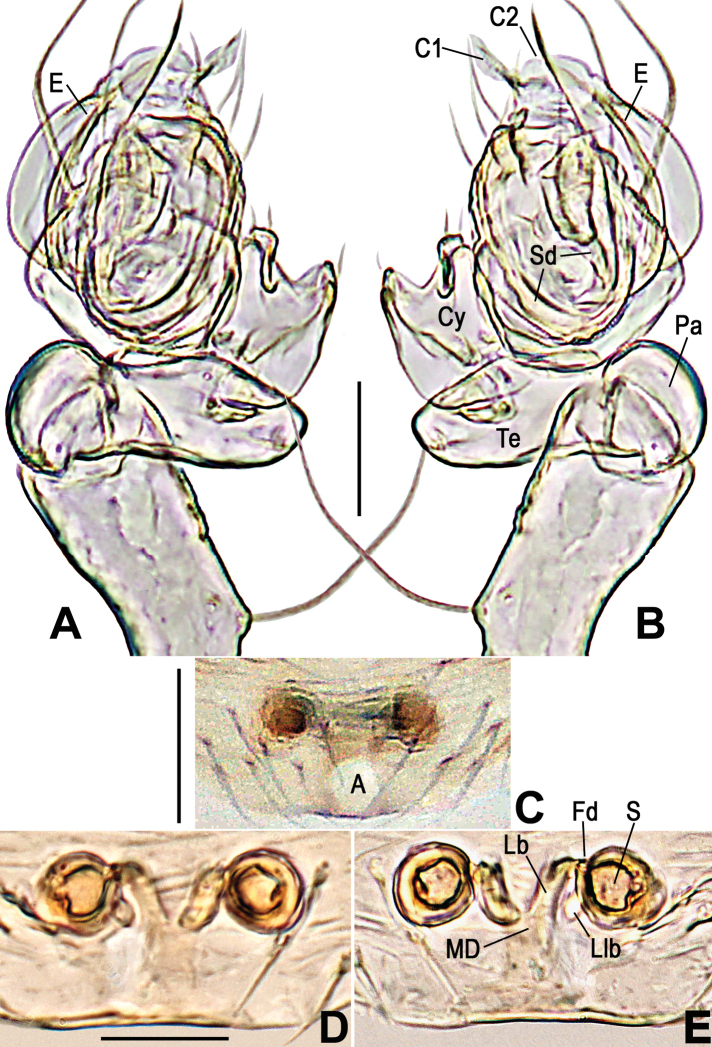
*Anapistulawalayaku* sp. nov. **A** male palp, prolateral **B** male palp, retrolateral **C** epigyne, ventral **D** vulva, ventral **E** vulva, dorsal. Abbreviations: A = epigynal atrium; C1 = anterior projection of conductor; C2 = posterior projection of conductor; Cy = cymbium; E = embolus; Fd = fertilization duct; Lb = lateral branch of the MD; Llb = distal lobe of lateral branch; MD = median duct of vulva; Pa = palpal patella; S = spermatheca; Sd = sperm duct; Te = palpal tibia. Scale bars: 0.10 (**A–E**).

Figs 3B, E, H, K , 5A–E , 8A–D

##### Type material.

***Holotype*** ♀ and ***paratypes*** 1♂ 6♀ (NHMSU-HA138), **China**: Yunnan Province, Nujiang Lisu Autonomous Prefecture, Lushui County, Daxingdi Township, Walayaku Cave (26.13198°N, 098.86149°E, 910 m), 10.VIII.2018, Y. Lin, Y. Li & Y. Shu leg.; 2♀ (NHMSU-HA106) from **China**: same data as for the holotype, 24.VI.2016, Y. Li leg. (NHMSU-HA106); 1♂, 1♀, 1 juv. (NHMSU-HA138), and 1♀ (NHMSU-HA106) used for sequencing, GenBank accession numbers given in Table 2, same data as for preceding.

##### Etymology.

The new species is named after the type locality; noun.

##### Diagnosis.

**Figure 6.**
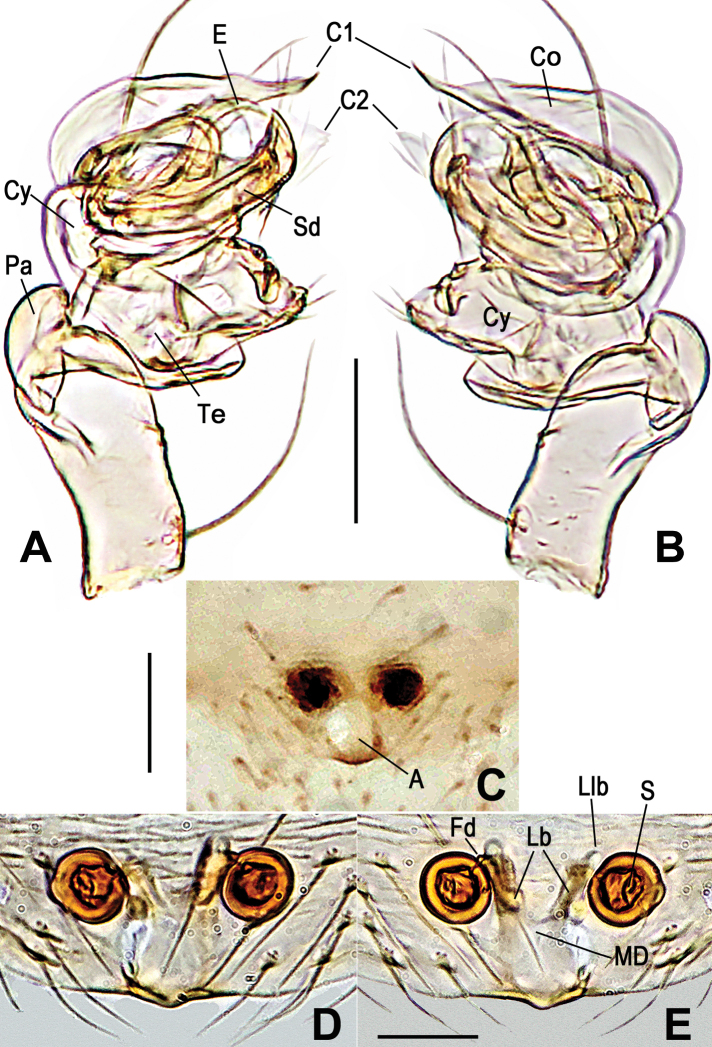
*Anapistulapanensis***A** male palp, prolateral **B** male palp, retrolateral **C** epigyne, ventral **D** vulva, ventral **E** vulva, dorsal. Abbreviations: A = epigynal atrium; Co = conductor; C1 = anterior projection of conductor; C2 = posterior projection of conductor; Cy = cymbium; E = embolus; Fd = fertilization duct; Lb = lateral branch of the MD; Llb = distal lobe of lateral branch; MD = median duct of vulva; Pa = palpal patella; S = spermatheca; Sd = sperm duct; Te = palpal tibia. Scale bars: 0.10 (**A–E**).

The male of *A.walayaku* sp. nov. is similar to that of *A.panensis* Lin, Tao & Li, 2013 by the relatively small bulb and the ventrally extended cymbium, but it differs by the short, blunt C2 (cf. Figs 5B, 8B to Figs 6B, 9B), the concave margin at the expanded part of the cymbium (cf. Figs 5A, B, 8A, B to Figs 6A, B, 9A, B) and the straight embolic tip (vs. bent) (cf. Figs 5A, 8A to Figs 6A, 9A). The female differs from most *Anapistula* species by the rounded atrium and the wide MD forming a Y-shape with the Lb (Figs 5C, E, 8C). It seems similar to *A.choojaiae* but can be distinguished by the narrower base of the MD and having an earlobe-shaped Llb (cf. Figs 5E, 8D to Rivera-Quiroz et al. 2021: figs 8d, 9c).

**Figure 7.**
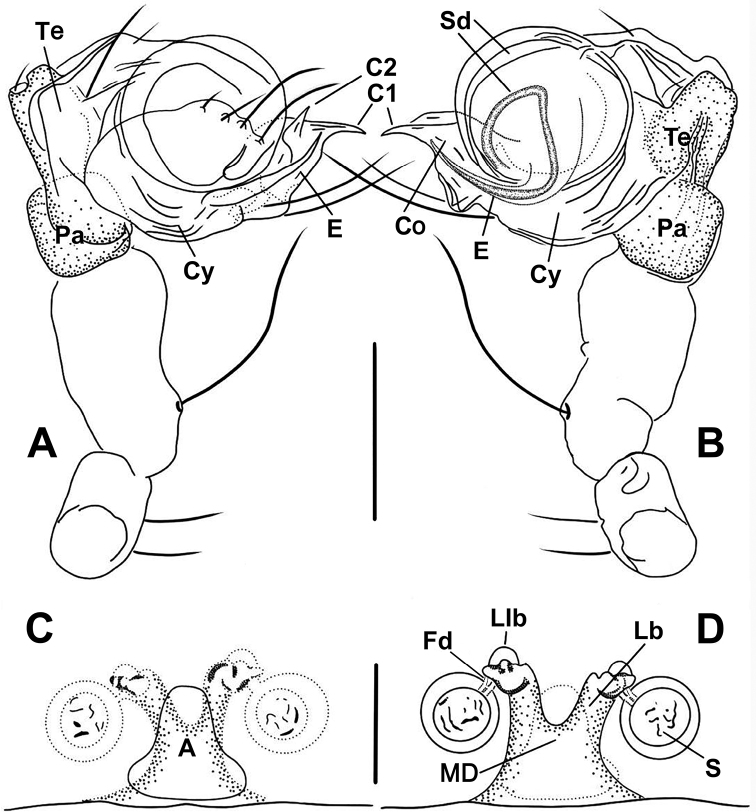
*Anapistulasanjiao* sp. nov. **A** male palp, prolateral **B** male palp, retrolateral **C** vulva, ventral **D** vulva, dorsal. Abbreviations: A = epigynal atrium; Co = conductor; C1 = anterior projection of conductor; C2 = posterior projection of conductor; Cy = cymbium; E = embolus; Fd = fertilization duct; Lb = lateral branch of the MD; Llb = distal lobe of lateral branch; MD = median duct of vulva; Pa = palpal patella; S = spermatheca; Sd = sperm duct; Te = palpal tibia. Scale bars: 0.10 (**A–D**).

**Figure 8.**
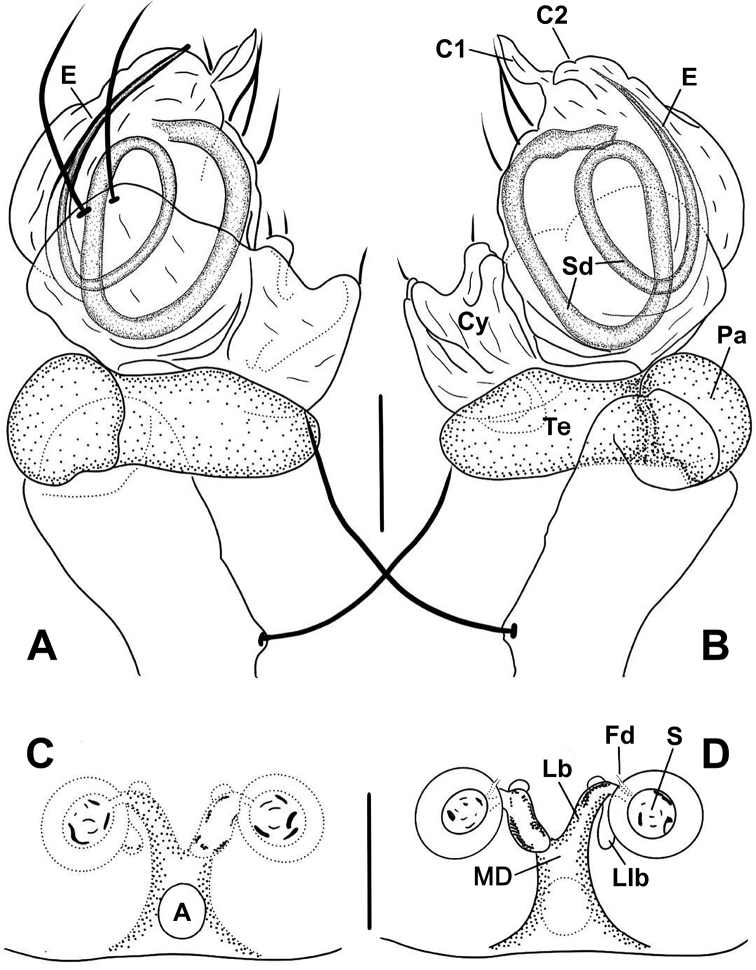
*Anapistulawalayaku* sp. nov. **A** male palp, prolateral **B** male palp, retrolateral **C** vulva, ventral **D** vulva, dorsal. Abbreviations: A = epigynal atrium; C1 = anterior projection of conductor; C2 = posterior projection of conductor; Cy = cymbium; E = embolus; Fd = fertilization duct; Lb = lateral branch of the MD; Llb = distal lobe of lateral branch; MD = median duct of vulva; Pa = palpal patella; S = spermatheca; Sd = sperm duct; Te = palpal tibia. Scale bars: 0.10 (**A–D**).

##### Description.

**Male**: Carapace nearly round in male, ovoid in female, pale centrally and pale brown marginally, smooth surface and two central short setae (Fig. 3B). Lateral eyes vestigial, barely visible (Fig. 3B). Chelicerae with two promarginal teeth. Labium rectangular, fused to sternum (Fig. 3E). Sternum peltate, as long as wide, smooth, slightly convex, with sparse, short setae (Fig. 3E). Legs same colour as carapace (Figs 3B, E). Abdomen unknown. Spinnerets and anal tubercle pale. Somatic measurements: body length unknown. Carapace 0.32 long, 0.28 wide, 0.24 high; sternum 0.20 long, 0.20 wide; length of legs: I 1.00 (0.28, 0.12, 0.24, 0.14, 0.22), II 0.82 (0.20, 0.10, 0.18, 0.12, 0.22), III 0.76 (0.18, 0.10, 0.16, 0.10, 0.22), IV 0.80 (0.20, 0.08, 0.20, 0.14, 0.18); leg formula I-IV-II-III.

***Palp***: small and weakly sclerotized. Femur swollen distally, with a long seta at retrolateral base. Patella short, as long as ½ length of tibia. Tibia contracted proximally, lacking setae. Cymbium with 4 retrolateral short and 3 dorsal long setae. Paracymbial rim concave, with 3 short setae (Figs 5B, 8B). Conductor sheet shaped, with two projections (C1 and C2), C1 sharp, C2 broad, blunt distally. Embolus long, needle shaped, located posterior to conductor, its end nearly reaches apex of C2. Sperm duct coiled ca 1.8 times inside bulb (Figs 5A, B, 8A, B).

**Female**: prosoma pear-shaped, palps absent, others as in male (Fig. 3H, K). Abdomen sub-spherical, yellow, dorsally darker than ventrally, cuticle with sparse, short setae and weakly sclerotized spots (Fig. 3H, K). Somatic measurements: body length 0.64, carapace 0.32 long, 0.24 wide, 0.24 high; sternum 0.24 long, 0.24 wide; length of legs: I 0.96 (0.30, 0.12, 0.20, 0.18, 0.16), II 0.86 (0.28, 0.08, 0.18, 0.18, 0.14), III 0.70 (0.18, 0.08, 0.16, 0.14, 0.14), IV 0.82 (0.20, 0.06, 0.22, 0.14, 0.20); leg formula I-IV-II-III; abdomen 0.44 long, 0.40 wide, 0.44 high.

***Epigyne***: flat, covered with sparse, long setae, without scape. Atrium nearly round, as broad as width of inner MD. Spermathecae spherical, separated by ca 1.2× their diameter, obviously sclerotized (Figs 3K, 5C, 8D). Lateral branch diverging from MD forming “Y” (Figs 5D, E, 8C, D), as wide as ½ MD, same long as ⅔ of MD. Lateral branch runs along dorsal surface of spermathecae and ends in a short, transparent Llb. Fertilization ducts very short, nearly invisible (Figs 5D, E, 8C, D).

##### Natural history.

This species was found in the crevices of stalagmites and stalactites in the dark zone of a cave.

##### Distribution.

China (Yunnan) (Fig. 10).

#### ﻿ Anapistula panensis

Taxon classificationAnimaliaAraneaeSymphytognathidae

Lin, Tao & S. Li, 2013

D65CF3D4-2C89-5E54-AE2B-17713D135C49

**Figure 9.**
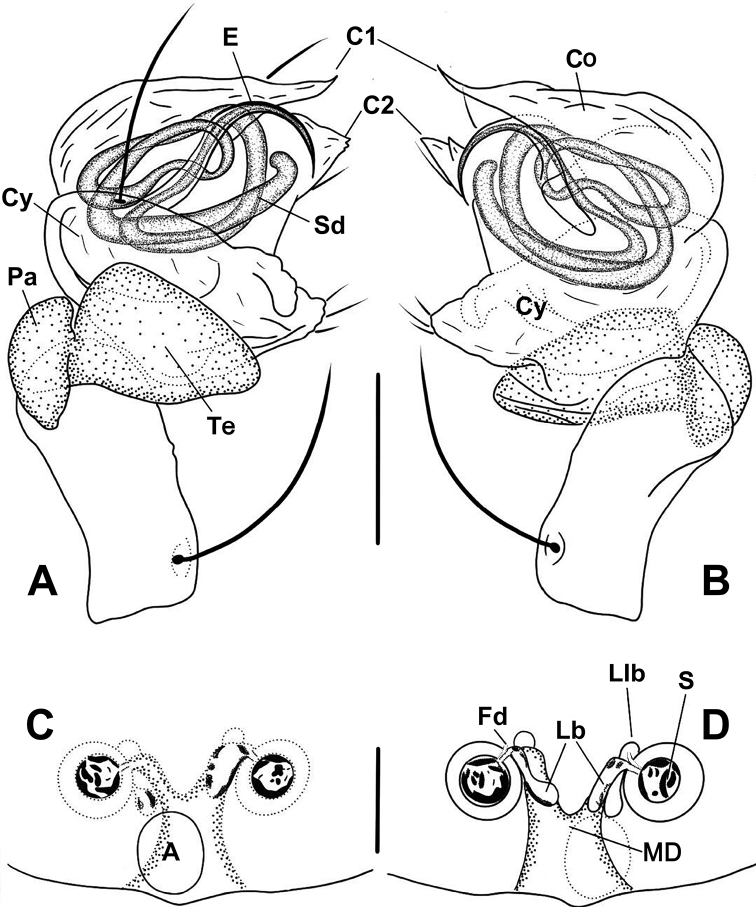
*Anapistulapanensis***A** male palp, prolateral **B** male palp, retrolateral **C** vulva, ventral **D** vulva, dorsal. Abbreviations: A = epigynal atrium; Co = conductor; C1 = anterior projection of conductor; C2 = posterior projection of conductor; Cy = cymbium; E = embolus; Fd = fertilization duct; Lb = lateral branch of the MD; Llb = distal lobe of lateral branch; MD = median duct of vulva; Pa = palpal patella; S = spermatheca; Sd = sperm duct; Te = palpal tibia. Scale bars: 0.10 (**A–D**).

Figs 3C, F, I, L , 6A–E , 9A–D

Anapistula panensis Lin, Tao & Li, 2013: 53, figs 1–5 (♂♀).

##### Type material.

***Holotype*** ♂ and ***paratypes*** 1♂ 50♀ (IZCAS) from **China**: Guizhou Province, Liupanshui City, Pan County, Zhudong Township, Shiliping Village, Shenxian Cave (25.62367°N, 104.75653°E, 1687 m), 15.IV.2007, J. Liu & Y. Lin leg. Examined.

##### Other material examined.

51♀ 18 juvs (NHMSU-HA020) from **China**: same data as type locality, 26.IV.2010, Y. Lin & Q. Zhao leg.; 1♂ 67♀ 20 juvs (NHMSU-QX003) same data as type locality, 24.VIII.2020, Y. Lin et al. leg.; 1♀ 1 juv (NHMSU-HA020) used for sequencing, GenBank accession numbers given in Table 2, same data as for preceding.

##### Diagnosis.

The male of *A.panensis* is similar to that of *A.choojaiae* in the shape of the palp and in having C1 and C2 roughly equal in length, but it differs by a narrower C1 and a wider C2, a longer embolus, and having three setae on the paracymbium (vs. two; cf. Figs 6A, B, 9A, B; Lin et al. 2013: figs 1, 2 with Rivera-Quiroz et al. 2021: figs 7c, 9a, b). The female differs from most *Anapistula* species by the Y-shaped epigynal median duct; it shares this character with *A.orbisterna*, *A.secreta*, *A.bifurcate*, *A.tonga*, *A.choojaiae*, *A.equatoriana*, *A.zhengi*, *A.sanjiao* sp. nov. and *A.walayaku* sp. nov. *Anapistulapanensis* differs from all of these by the width and length of the MD, the length of the lateral branches, and the shape and relative size of the atrium (Figs 6C–E, 9C, D and Lin et al. 2013: figs 3, 4 vs. Forster and Platnick 1977: fig. 19; Harvey 1998: figs 9, 19; Rivera-Quiroz et al. 2021: figs 8d, 9c; Dupérré and Tapia 2017: fig. 33; Lin et al. 2013: figs 8, 9).

##### Redescription.

**Male**: habitus as in Fig. 3C, F. Body yellow. Legs pale yellow. Carapace nearly round, cephalic area moderately elevated. Four vestigial eyes in diads. Chelicerae distinctly sclerotized and fused basally, concave at inner margins, with two promarginal teeth. Endites as long as wide. Labium rectangular, length ca equal to ⅓ of width, fused to sternum. Sternum flat, with sparse setae, truncated posteriorly. Femur I and II swollen retrolatero-basally, tiny serrations and granulations on surface. Patellae I–IV each with a distal-dorsal seta. Abdomen ovoid dorsally, higher than long, covered with sparse, grey, long setae, posterior expanded beyond spinnerets. Colulus absent. Somatic measurement: body length 0.56, carapace 0.28 long, 0.28 wide, 0.24 high; sternum 0.20 long, 0.24 wide; abdomen 0.32 long, 0.28 wide, 0.36 high; length of legs: I 1.18 (0.34, 0.14, 0.26, 0.14, 0.30), II 0.96 (0.28, 0.12, 0.18, 0.12, 0.26), III 0.80 (0.20, 0.10, 0.16, 0.10, 0.24), IV 0.98 (0.30, 0.12, 0.18, 0.16, 0.22); leg formula I-IV-II-III.

***Palp***: small and weakly sclerotized. Femur slightly swollen distally, with a long seta at retrolateral base. Patella short, semilunar shaped. Tibia contracted proximally, broad distally. Cymbium transparent, with 7 retrolateral short and 2 dorsal long setae. Conductor sheet shaped, with two projections (C1 and C2), C1 sharp, C2 lamellar, nearly invisible. Embolus short, needle shaped, posterior to conductor. Sd coiled ca 2 times inside bulb (Figs 6A, B, 9A, B).

**Female**: habitus see Fig. 3I, L. Carapace darker yellow than abdomen. Palps absent, others as in male. Somatic measurements: body length 0.68, carapace 0.28 long, 0.28 wide, 0.24 high; sternum 0.20 long, 0.18 wide; abdomen 0.50 long, 0.52 wide, 0.52 high; length of legs: I 1.18 (0.36, 0.14, 0.24, 0.20, 0.24), II 1.08 (0.30, 0.14, 0.24, 0.18, 0.22), III 0.82 (0.18, 0.12, 0.16, 0.16, 0.20), IV 1.12 (0.30, 0.14, 0.26, 0.18, 0.24); leg formula I-IV-II-III.

***Epigyne***: flat, without scape. Atrium ovoid, narrower than space between spermathecae. Spermathecae spherical, separated by ca 1.3× their diameter, obviously sclerotized (Figs 3L, 6C, 9C). Lateral branches diverging from MD, forming Y-shape (Figs 6D, E, 9C, D), as wide as ⅓ of MD, as long as MD (Fig. 6E; Lin et al. 2013: figs 3, 4). Fertilization ducts very short, translucent, nearly invisible, Llb appear as tiny bumps distally on lateral branches (Figs 6D, E, 9C, D).

##### Natural history.

This species spins a small, flat circular web in the crevices of stalagmites or stalactites in caves.

##### Distribution.

**Figure 10.**
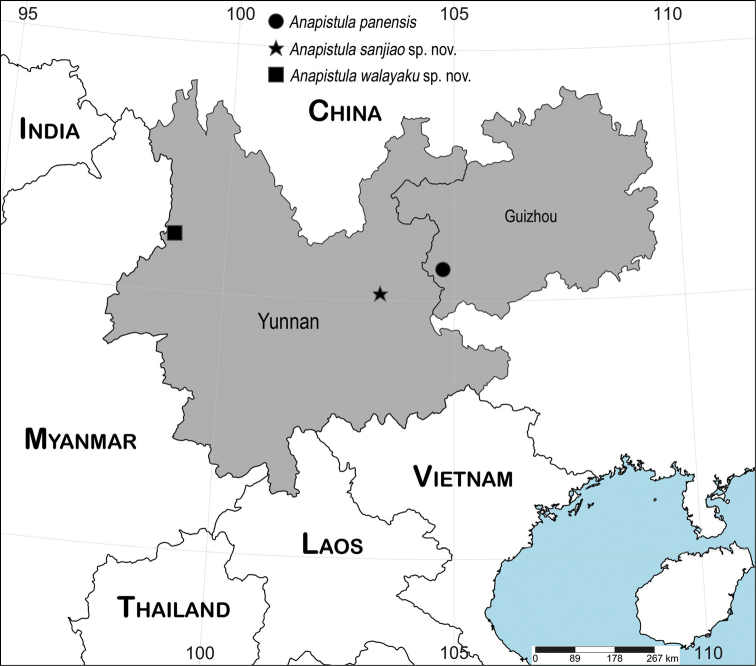
Distribution records of three Chinese cave-dwelling *Anapistula* species.

China (Guizhou) (Fig. 10).

## ﻿Discussion

The taxonomy of symphytognathoids is inadequate due to their small size and difficulty in collection. However, the worldwide species diversity of this family has increased from 37 species in eight genera to 98 species in ten genera in the past 20 years (WSC 2022). Recent studies have reported 48 species from Asia (Ono 2002; Tong and Li 2006; Lin and Li 2009; Lin et al. 2009, 2013; Miller et al. 2009; Shinkai 2009; Miller et al. 2014; Lin 2019; Li et al. 2020, 2021; Rivera-Quiroz et al. 2021) and 11 species from South America (Rheims and Brescovit 2003; Brescovitet al. 2004; Rubio and González 2010; Dupérré and Tapia 2017).

The symphytognathoids were first proposed as a morphological group by Griswold et al. (1998), who postulated that this spider group consisted of the monophyletic families Theridiosomatidae, Mysmenidae, Symphytognathidae and Anapidae. However, the monophyly of Symphytognathidae and its relationships to the other three families are complex and inconsistent in different phylogenetic studies. Symphytognathidae has been used repeatedly as a representative clade to test the phylogenetic relationships of specific groups, such as “symphytognathoids” (Rix et al. 2008; Lopardo et al. 2011; Feng et al. 2019), the Orbiculariae (Lopardo and Hormiga 2008; Fernández et al. 2014; Rivera-Quiroz et al. 2021) and all Araneae (Dimitrov et al. 2012; Wheeler et al. 2017; Kulkarni et al. 2020) using different molecular approaches and analyses. However, these studies were limited by missing data, including species and markers. The taxonomic status and validity of most symphytognathoid genera and species have not been tested with molecular phylogenetic methods, and the systematics of the family Symphytognathidae is pending.

In this study, we tested the monophyly of Symphytognathidae, but support values were low, probably due to the limited number of representative taxa. Our MP analysis failed to recover the monophyly of Anapidae. In contrast to the results of Rivera-Quiroz et al. (2021), our MP and BI analyses resolved the position of the Micropholcommatinae as within the Anapidae.

## Supplementary Material

XML Treatment for Anapistula

XML Treatment for Anapistula sanjiao

XML Treatment for Anapistula walayaku

XML Treatment for Anapistula panensis
